# P-883. A Prospective Audit of Discharge Antibiotic Prescribing: Patterns, Indications, and Stewardship Insights from a Tertiary Care Center

**DOI:** 10.1093/ofid/ofaf695.1091

**Published:** 2026-01-11

**Authors:** Giris Sharma, Libis Linga Siva Subbu Raj, R Jashwanth Jayannth, Sathya Narayanan, E D Dinesh

**Affiliations:** Tamilnadu Dr.MGR Medical University, Chennai, Tamil Nadu, India; Tamilnadu Dr.MGR Medical University, Chennai, Tamil Nadu, India; Tamilnadu Dr.MGR Medical University, Chennai, Tamil Nadu, India; Tamilnadu Dr.MGR University, Chennai, Tamil Nadu, India; Tamilnadu Dr.MGR Medical University, Chennai, Tamil Nadu, India

## Abstract

**Background:**

Antibiotic prescribing at hospital discharge significantly contributes to antimicrobial exposure, often extending therapy beyond the clinical need. Studies reveal that many discharge prescriptions are unnecessary or prolonged, increasing antimicrobial resistance and adverse outcomes. While inpatient stewardship has advanced, discharge practices that remain Unnoticed. This study analyzes discharge antibiotic patterns categorized as AWaRe groups, empirical, definitive, or prophylactic and their duration. By finding where antibiotic use could be better managed, we aim to support better prescribing during care transitions and reduce resistance in the community.

**Figure:**
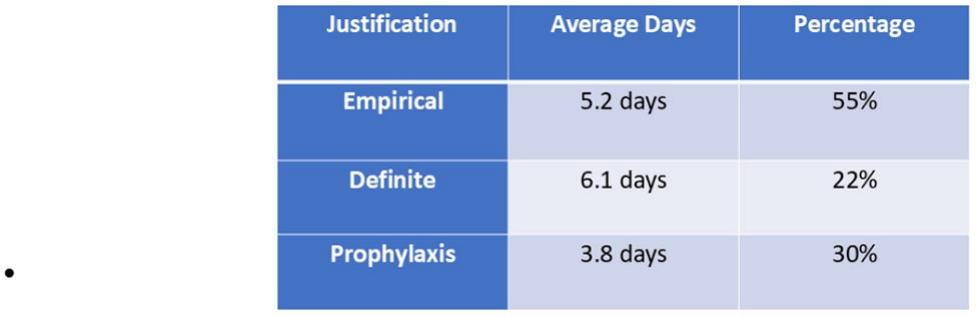
Antibiotic Use at Discharge: Clinical Justification and Average Duration This table shows the average duration of antibiotics used at discharge, categorized by clinical justification: empirical, definitive or prophylactic.

**Figure:**
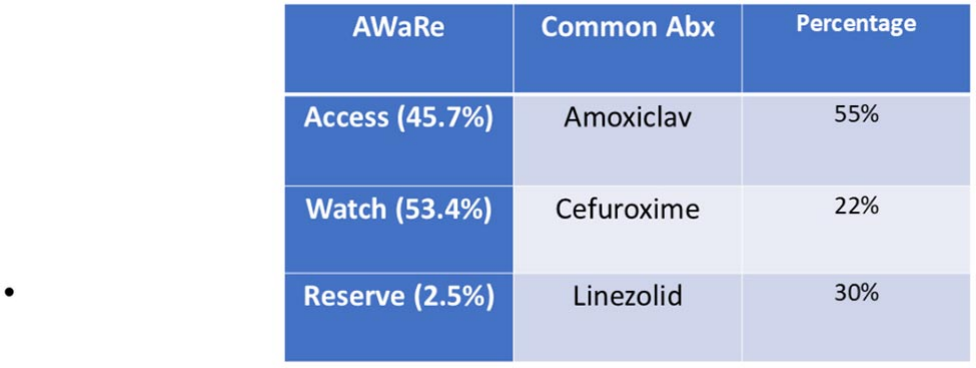
AWaRe Classification of Discharge Antibiotics This table shows the most common antibiotics used at discharge, grouped by WHO AWaRe categories and their usage percentages.

**Methods:**

A prospective audit was conducted at a South Indian tertiary hospital from March to June 2024. Patients prescribed antibiotics at discharge were included, with exclusions for those under 18, discharged against medical advice, or not receiving antibiotics. Data collection focused on antibiotic type, administration route, WHO AWaRe classification and prescribing rationale (empirical, definitive, or prophylactic).

**Results:**

Out of 1,607 discharge summaries audited, 782 (48.7%) included antibiotic prescriptions. Most were oral (79%), with limited IV use (16%). Empirical therapy was the most frequent indication (55%; mean duration: 5.2 days), followed by prophylactic use (30%; mean: 3.8 days) and definitive therapy (22%; mean: 6.1 days). Antibiotics prescribed fell into the following AWaRe categories: Access (45.7%), Watch (53.4%), and Reserve (2.5%). A progressive improvement in antibiotic classification and prescribing appropriateness was observed over the study period.

**Conclusion:**

This study highlights significant empirical antibiotic use at discharge. Strengthening stewardship during care transitions can improve prescribing quality, reduce unnecessary exposure, and the risk of antimicrobial resistance.

**Disclosures:**

All Authors: No reported disclosures

